# Validity of a Short Food Frequency Questionnaire Assessing Macronutrient and Fiber Intakes in Patients of Han Chinese Descent with Type 2 Diabetes

**DOI:** 10.3390/ijerph15061142

**Published:** 2018-06-01

**Authors:** Meng-Chuan Huang, Kun-Der Lin, Hung-Jiun Chen, Yu-Ju Wu, Chiao-I Chang, Shyi-Jang Shin, Hsin-Chia Hung, Chien-Hung Lee, Ya-Fang Huang, Chih-Cheng Hsu

**Affiliations:** 1 Department of Nutrition and Dietetics, Kaohsiung Medical University Hospital, Kaohsiung 80705, Taiwan; mechhu@kmu.edu.tw (M.-C.H.); 1010213@kmuh.org.tw (H.-J.C.); erin@cc.kmu.edu.tw (Y.-J.W.); 2 Graduate Institute of Medicine and Department of Public Health and Environmental Medicine, College of Medicine, School of Medicine, Kaohsiung Medical University, Kaohsiung 80705, Taiwan; s370002000@yahoo.com.tw; 3 Department of Internal Medicine, Kaohsiung Municipal Ta-Tung Hospital, Kaohsiung Medical University, Kaohsiung 80145, Taiwan; 890073@ms.kmuh.org.tw; 4 Division of Endocrinology and Metabolism, Department of Internal Medicine, Kaohsiung Medical University Hospital and College of Medicine, Kaohsiung 80705, Taiwan; sjshin@cc.kmu.edu.tw; 5 Department of Oral Hygiene, College of Dental Medicine, Kaohsiung Medical University, Kaohsiung 80705, Taiwan; hhung2006@gmail.com; 6 Department of Public Health, College of Health Sciences, Kaohsiung Medical University, Kaohsiung 80705, Taiwan; cnhung@kmu.edu.tw; 7 Institute of Population Health Sciences, National Health Research Institutes, 35 Keyan Road, Zhunan 35053, Miaoli County, Taiwan; avon@nhri.org.tw; 8 Department of Health Services Administration, China Medical University, Taichung 40402, Taiwan; 9 Department of Family Medicine, Min-Sheng General Hospital, Taoyuan 33044, Taiwan

**Keywords:** carotenoids, 24-h dietary recall, food frequency questionnaires, type 2 diabetes, validity

## Abstract

Few food frequency questionnaires (FFQs) have been developed to assess diet in diabetes patients. This cross-sectional study examined the validity of a 45-item FFQ assessing the intake of macronutrients against three 24-h dietary recalls (24-HDRs) in Taiwan, and compared vegetable and fruit intakes with carotenoid biomarkers. We recruited 126 adults with type 2 diabetes who completed the FFQ and three 24-HDRs administered by a registered dietitian. We measured plasma carotenoids (α-carotene, β-carotene and lutein) in 71 subjects. Partial Pearson correlation coefficients derived from the FFQs and three 24-HDRs and adjusted for energy were of 0.651, 0.587, 0.639 and 0.664 for protein, fat, carbohydrate and fiber, respectively. Cross-classification analysis revealed that 71.5–81% of the macronutrients and fiber were categorized into the same or adjacent quartiles by the FFQ and 24-HDRs. Bland–Altman plots revealed good agreement for energy/macronutrients/fiber across the range of intakes. Multiple linear regression of backward elimination revealed that tertile levels of dark- or light-colored vegetables obtained by the FFQ were significantly associated with plasma α-carotene and β-carotene, but not lutein. Fruit consumption did not correlate with carotenoid biomarkers. In conclusion, this short FFQ provided a valid assessment of macronutrients and fiber intake in type 2 diabetes patients. Vegetable consumption estimated by the FFQ corresponded to plasma α-carotene and β-carotene concentrations.

## 1. Introduction

Basing their estimates on reported diabetes prevalence figures for 111 countries, the International Diabetes Federation (IDF) reported that 415 million people had diabetes in 2015. The number of people with diabetes aged 20–79 years is predicted to rise to 642 million by 2040 [1]. Diabetes caused 1.3 million deaths in 2010, twice as many as in 1990 [2]. In addition, diabetes-associated complications, including retinopathy, nephropathy, and cardiovascular diseases pose significant challenges to individuals and healthcare systems [3]. In Taiwan, the prevalence of diagnosed type 2 diabetes increased significantly from 5.79% in 2000 to 8.30% in 2007 [4]. The total healthcare cost per diabetes patient is about 2.8 times higher than for those without diabetes [5].

Management of diabetes requires both pharmacological interventions and lifestyle changes, including dietary management [6]. Dietary assessment is important for health promotion efforts and disease prevention, as well as individual treatment plans. In clinical settings, questionnaires assessing diet not only need to be valid and reliable, but they also need to be easy and quick to administer. Food frequency questionnaires (FFQs) are used to estimate individual perception of standard food intake over a defined period such as a year or several months [7]. Compared to other means of dietary assessment, which often require much training, FFQs are less expensive and less difficult to administer despite the crude information they are thought to provide [8]. Multiple dietary recalls [9], food records [10,11], or biomarkers [12,13] are generally accepted to provide more accurate estimates of nutrient intakes, and they often serve as reference when validating newly developed FFQs.

Studies using various FFQs targeting diabetes have reported an association between intakes of various nutrients or foods and risk of diabetes or its complications. FFQs were used to evaluate the risks associated with intake of red meat, unprocessed red meat, processed meat and poultry in healthy 45–75-year-old Japanese men and women [14]. They were also used to evaluate the development of diabetes risks associated with protein intake in healthy adults in Australia [15]. FFQs were used to evaluate the risks of intake of various macronutrients (carbohydrate, fat, saturated fat, dietary fiber, and glycemic load), with data derived from self-reports of dietary intake as well as circulating n-3 polyunsaturated fatty acids in blood in diabetes cases in Europe [16]. In China, some FFQs have been developed for food intake studies of pregnant women [17,18] and adults in a community-based nutrition and health survey [19]. In Taiwan, however, investigations exploring the associations between intake of nutrients or food items and risks of chronic diseases have been limited by the availability of valid FFQs. In Taiwan, FFQs have been used to study healthy populations, including college students [20], the elderly [21], adults [22], and adult vegetarians [23], but there are none targeting people with diabetes, though diabetes has gradually become a public health threat.

In this study, we evaluated the validity of a FFQ used to estimate macronutrient intakes in adult patients with type 2 diabetes. Energy and macronutrient intake as assessed by a single FFQ was compared against intake assessed by three 24-h dietary recalls (24-HDRs). For comparison and further validation, we also studied the correlation between FFQ-collected consumption frequencies of different food groups and carotenoids as antioxidant biomarkers. 

## 2. Materials and Methods

### 2.1. Study Design and Population

The study subjects were chosen from a cohort of type 2 diabetes patients enrolled in an ongoing intervention study investigating the effect of tight blood pressure control on reduction of renal risk for type 2 diabetes mellitus (the BP4DM study, Clinicaltrials.gov NCT03477786). The original study recruited 30- to 75-year-old type 2 diabetes patients with hypertension (systolic or diastolic blood pressure >140/90 mmHg) who had registered to participate in a multidisciplinary diabetes shared-care program. Subjects not eligible at baseline recruitment included those with type 1 diabetes, gestational diabetes, pregnant women, hemoglobin A1c (HbA1c) >10%, eGFR (estimated glomerular filtration rate) <30 mL/min/1.73 m^2^, albuminuria > 300 mg/g, presence of non-diabetes-associated albuminuria, kidney stones >0.5 cm, or a history of myocardial infarction as well as those with a history of cerebrovascular events, foot amputation, dialysis, cirrhosis, or cancer under active treatment within 3 years. The baseline recruitment period for this study was between October 2013 and July 2015; we cross-sectionally recruited 150 of the subjects in the original study to participate in interviews with a single registered dietitian responsible for collecting of all dietary data. Because this was a validation study, we excluded patients who reported implausible energy intakes (<500 or >7000 kcal/day) or who had missing demographic and dietary data, leaving us with 126 patients, all of whom completed a food frequency questionnaire (FFQ) and 24-h dietary recalls. We then randomly selected 71 subjects whose blood specimens we used to measure plasma carotenoids. The 24-h dietary recalls were taken within a week of the FFQ. The FFQ was administered and plasma was collected at the second annual visit, not at baseline. This study was approved by the Ethics Committee of the Kaohsiung Medical University and Taiwan’s National Health Research Institutes. Written informed consent was obtained from each subject.

### 2.2. Collection of Demographic and Anthropometric Data

Age, sex, duration of diabetes, educational status, and lifestyle factors were obtained using a structured questionnaire administered at the same time patients received routine checkups, every six months. Lifestyle factors were evaluated by asking whether they currently smoked (yes, no), whether they drank alcohol at least once a week in the previous six months (yes, no), and whether they had had any exercise within the last two weeks (yes, no). Body mass index (BMI), waist circumferences, and blood pressure measurements were measured by trained research nurses or assistants.

### 2.3. Assessment of Dietary Intakes Using FFQ and Three 24-HDRs

Dietary intake was surveyed using a 45-question semi-quantitative food frequency questionnaire (FFQ) structured to evaluate frequency of food group consumption, serving sizes, and some eating habits. Subjects were asked how frequently they consumed items belonging to certain food groups over the previous six months, with nine frequency options ranging from “almost never” to “four to six times per day”. Consumption frequencies of certain food groups (Chinese staple foods, bread and cereals, root vegetables, light- or dark-colored vegetables, fresh fruits, canned juices, marine or pound fish, red or white meats, eggs, soy products, milk, dairy products, etc.) were expressed as usual portion sizes consumed per day, week, or month. All of the above food groups were then converted to weekly equivalents, and then the daily energy intake for each participant was estimated. Details of the dietary assessment using this FFQ have been previously reported in studies correlating certain dietary patterns and risk of renal diseases and metabolic alterations in type 2 diabetes [24,25].

A registered dietitian conducted an interview with each participant to obtain three 24-HDRs. The three days assessed were two weekdays and one weekend day. Detailed records of food items, cooking methods, time of eating, and eating places were recorded. Intake was assessed using commonly used units such as bowls, spoons, and the use of food models or picture illustrations to help estimate amounts of food consumed. Analysis of food nutrients was also performed by the same registered dietitian using nutrient analysis software (E Kitchen Business Corporation, Taichung, Taiwan). The daily intakes of foods were calculated and analyzed as daily energy (kcal/day), protein (g/day), lipid (g/day), carbohydrate (g/day), and fiber (g/day) intake. The average nutrient intake of each subject was calculated as an average of three days.

### 2.4. Measurement of Clinical Parameters

Overnight fasting venous blood samples were taken at least 8–10 h after the last meal, and spot urine samples were collected, kept at 2–8 °C, and sent to a central laboratory (Union Clinical Laboratory, Taipei, Taiwan) certified by The College of American Pathology and US Commission on Office Laboratory Accreditation. Fasting plasma glucose, cholesterol, triglyceride, low-density lipoprotein cholesterol (LDL-C), high-density lipoprotein cholesterol (HDL-C), uric acid, blood urea nitrogen (BUN), and creatinine were analyzed using an auto-analyzer (ADVIA 1800, Siemens Healthcare Diagnostics, New York, NY, USA). Hemoglobin A1c (HbA1c) was measured by high-performance liquid chromatography (Variant II; Bio-Rad Laboratories, Hercules, CA, USA). Urinary and serum creatinine were measured by the Jaffe reaction method (ADVIA 1800, Siemens Healthcare Diagnostics, New York, NY, USA). Microalbuminuria and albumin creatinine ratios were calculated using Polyethylenglycol-enhanced immunoturbidimetric (ADVIA 1800, Siemens Healthcare Diagnostics, New York, NY, USA).

Hypertension was defined in a subject if he or she had a measured blood pressure of ≥140/90 mmHg or the subject self-reported use of anti-hypertensive medications (yes, no). Fasting (≥8 h) hemoglobin A1C (HbA1C) was measured using high-performance liquid chromatography (Tosoh, Kobe, Japan), and serum triglycerides were measured using an auto-analyzer (Beckman Coulter, Fullerton, CA, USA). 

### 2.5. Measurement of Plasma Carotenoids

After overnight fasting, plasma was analyzed for carotenoids by high-pressure liquid chromatography (HPLC) based on a modified procedure reported previously [26]. Briefly, before analysis, the samples were thoroughly mixed with ethanol and hexane, and then centrifuged at 10,000 rpm for five minutes. The hexane layer was collected, evaporated under nitrogen and finally dissolved in a mobile phase in a high pressure liquid chromatography (HPLC) system. The mobile phase consisted of a solvent system with 68:22:7 (v/v/v) acetonitrile tetrahydrofuran methanol and a 1% (v/v) ammonium acetate solvent system. The separation module (Waters 2695; Waters Corp., Milford, MA, USA) was equipped with The Waters Novapak C18 column (150 × 3.9 mm), a programmable multi-wavelength detector equipped with a Dual λ Absorbance Detector (Waters, Milford, MA, USA) and a Scanning Fluorescence Detector (Waters, Milford, MA, USA). The detection wavelength for carotenoid analysis was set at 450 nm. The column was prepared with a pre-column module (Guard-Pak Pre-column; Waters Corp.) and all samples were pre-filtered through a 0.45-μm filter. The analytical procedures used in this study have been modified from a previously reported program [27].

### 2.6. Statistical Analysis

A one-sample Kolmogorov–Smirnov test was used to examine the normality of energy, macronutrients, and fiber. These data were log transformed to improve normalization of the skewed distribution before analysis. For validation, differences between the energy and macronutrient intake values derived from the FFQ or the three 24-HDRs were determined using paired *t* test. The Pearson correlation was used to determine correlations between energy and macronutrient intake derived from the above two dietary assessment tools. Partial correlations were calculated with data adjusted for energy intake according to residual methods [28]. For agreement analysis, we further examined the quartile distribution of energy-adjusted nutrient intakes between the two methods in order to classify whether they fell into the same, adjacent, one-quartile-apart, or far apart quartiles. The Bland–Altman method [29] was also used to assess agreement between nutrient intake values obtained using two dietary assessment tools.

Simple linear regression was employed to test the trends of plasma carotenoids concentrations across tertile intake levels of different food groups. Independent associations between tertile intake levels of light-green vegetables, dark-green vegetables, or fruits obtained from the FFQ and carotenoids were tested using multiple linear regression of backward elimination adjusting for potential confounding factors, including age, gender, energy intake based on the FFQ, diabetes duration, education, hemoglobin A1c (<6.5%, 6.5–9% and >9%), and use of vitamin supplements (multivitamins, vitamin A or vitamin C).

All statistical operations were performed using SPSS Version 22.0 (SPSS Inc., Chicago, IL, USA). A *p* < 0.05 was considered significant.

## 3. Results

### 3.1. Characteristics of Type 2 Diabetes Patients

The demographic and clinical characteristics of type 2 diabetes patients (*n* = 126) are shown in Supplementary Table 1. The participants were predominantly below 65 years old (53.2%), had diabetes for less than ten years (47.6%), had high school-level education or higher (52.4%), were past smokers (65.9%), and ate out >1 times per day (52.4%). Subjects had a mean BMI and waist circumference of 27.0 (±4.1) kg/m^2^ and 92.3 (±10.7) cm, respectively. Participants had mean fasting glucose and HbA1c of 139.6 (±45.4) mg/dL and 7.0 (±1.0)%, and more than 60% of them had HbA1c ≥6.5%. Systolic and diastolic blood pressures were 124.0 (±76.1) and 65.5 (±71.8) mmHg, respectively. The mean values for blood lipids were as follows: triglycerides (164.8 ± 228.5 mg/dL), total cholesterol (167.9 ± 30.4 mg/dL), LDL-C (92.0 ± 24.4 mg/dL), and HDL-C (46.2 ± 14.6 mg/dL) (Supplementary Table S1).

### 3.2. Validation of the FFQ to Assess Macronutrients

Table 1 shows the mean of daily energy, macronutrient and fiber intakes estimated by our FFQ compared with the mean values obtained by three 24-HDRs (Table 1). Daily intakes of energy and all three macronutrients (protein, fat and carbohydrate) and fiber obtained from the FFQ were compared with those obtained using 24-HDRs. The Pearson correlation coefficients for the crude dietary data ranged from 0.501 (energy) 0.574 (protein), 0.514 (fat), 0.438 (carbohydrate), and 0.666 (fiber). After adjustment for total energy intake, correlations (*r* = 0.587–0.664, all *p* < 0.001) in three macronutrients and fiber were all improved using residual methods.

In a cross-classification of nutrients, we classified the energy-adjusted macronutrient intakes into quartiles (Table 2). The agreement rates for the same or adjacent quintile classifications of our participants ranged from 71.5% (fat) to 81% (fiber) in the same or adjacent quintile classifications. Extreme quartile misclassification ranged from 1.6% (fiber) to 8.7% (carbohydrate).

**Figure 1:**
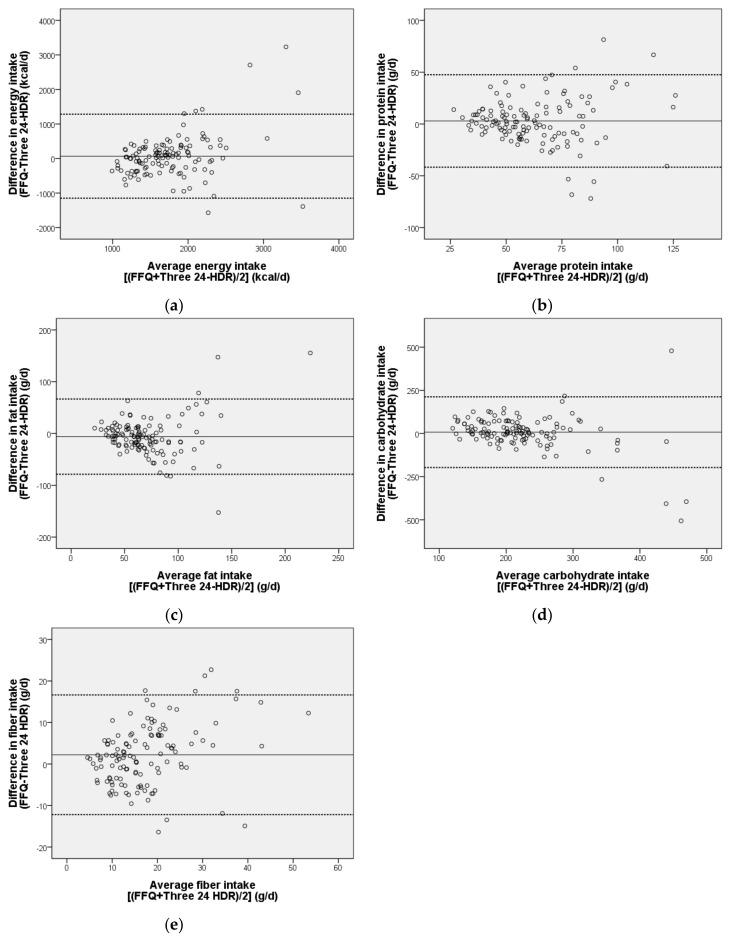
Bland–Altman plots showing the agreement with respect to (**a**) energy, (**b**) protein, (**c**) fat, (**d**) carbohydrate and (**e**) fiber intakes assessed using a short FFQ and 24-HDRs in type 2 diabetes patients in Taiwan. Solid lines indicate the mean of the differences, and the dashed lines indicate the lower and upper 95% limits of agreement.

The Bland–Altman plot analysis graphs (Figure 1) show a fair agreement between FFQ and 24-HDR methods in their assessments of energy, macronutrients, and fiber. The proportion of plots being outside the lower and upper limits of agreement ranged from 4.8% to 6.3%.

### 3.3. Concentrations of Plasma Carotenoids among Tertile Levels of Food Groups Intakes

Table 3 shows a summary result of our univariate analysis of association between the plasma carotenoid concentrations (*n* = 71) and tertile intake levels of select food categories used in the FFQ (Table 3). It appears that subjects with increased intake levels of light/dark vegetables and marine fish had increasing trends in plasma α- and β-carotene (all *p* for trend < 0.05), but not lutein. On the other hand, three food groups, including fried foods (*p* for trend = 0.022), egg products (*p* for trend = 0.012), and fermented products (*p* for trend = 0.044), were negatively associated with plasma α-carotene (data not shown). All other food groups in our questionnaire, including fruits and fruit juices, did not correlate with any of the three carotenoids.

### 3.4. Independent Association between Tertile Intake Levels of Selected Food Items and Carotenoids

Independent association between vegetable or fruit food groups and three carotenoids were analyzed using backwards elimination methods (Table 4). After adjustment for age, gender, FFQ-derived total energy intake, diabetes duration, and education, increased intake of light or dark vegetable food group remained significantly (*p* = 0.009–0.035) associated with plasma α- carotene or β-carotene. Dark- or light-colored vegetables were not correlated with lutein, being eliminated from each model using regression by backwards elimination. Only combining dark and light vegetables together was found to be marginally correlated with lutein (*p* = 0.090). The food group of fresh fruits or fruit juices did not appear to correlate to any of the three carotenoids when eliminated from all models.

## 4. Discussion

This study found our short 45-item FFQ to be a valid assessment of intake of various macronutrients and fiber in adult patients with type 2 diabetes in Taiwan. The macronutrient and fiber intake values estimated using our 45-item FFQ were found to be in high agreement with the results of repeated three 24-HDRs, with coefficient of correlations ranging from 0.587 to 0.664 after adjusting for energy. It was found that 71.5–81% of subjects were correctly classified into the same or adjacent quartiles for three macronutrients and fiber. Additionally, Bland–Altman plotting also revealed an acceptable level of agreement between the two methods.

Validation studies are usually performed for newly designed food intake questionnaires to evaluate whether they measure what they are designed to measure or to assess the degree to which they agree with a ‘gold standard’ or other methods of measuring diet. Thus, it is important to compare the new FFQ against an appropriate well-accepted reference method [30]. One review studying validation of FFQs found that 22% of the validation studies they reviewed used multiple 24-h recalls as reference measures, 25% used weighed records, 26% used food diaries, 6% used diet history questionnaires, and 12% used other FFQs [8]. The energy-adjusted coefficients of correlations of three macronutrients against multiple 24-HDRs in some previous investigations have been reported to range from 0.30 to 0.54 in Canadian adults [9], 0.43 to 0.85 in blacks of American and Caribbean origin and Caucasians [31], 0.60 to 0.76 in participants studied in the European Prospective Investigation into Cancer and Nutrition (EPIC) Study in Potsdam and Germany [32], and 0.49 to 0.53 in female adolescents in China [33]. The partial Pearson correlation coefficients of three macronutrients (0.587–0.651) observed in our current investigation are consistent with those reported in the above ethnic populations.

FFQs and 24-HDRs share some similar sources of errors, including reliance on memory and perception of portion sizes [7,8], though one is reliant on long-term memory and the other on short-term memory. The FFQ can be self-administered and contains close-ended questions, while 24-HDRs are administered by interviewers asking open-ended questions. These differences suggest that the errors are sufficiently independent and that the 24-HDR can be used with some confidence to validate an FFQ [34]. The use of correlation analysis to validate a new measurement technique with an established one has frequently been questioned because such analysis does not measure agreement but assesses the extent of association between two variables [35]. Therefore, we used cross-classification into quartiles of intake and Bland–Altman plots to better clarify the extent of agreement between the two methods we used. With regard to its classification of nutrient intake into total energy, macronutrients, and fiber, the FFQ we developed was found to classify a relatively high proportion of subjects (71.5–81%) into the same or adjacent category as did the three 24-HDRs. Only a small number of individuals (1.6–8.7%) were misclassified (Table 3). As can be seen in our Bland–Altman plots (Figure 1), only 4.8% of the values fell outside the lower and upper 95% limits (mean ± 1.96 SD) of agreement for fat, 4.8% for carbohydrate, 6.3% for protein, 6.3% for fiber, and 6.3% for energy (Figure 1).

Fat and fiber intakes estimated by our FFQ were observed to be approximately 20% higher than those estimated by the three 24-HDRs. These discrepancies can be partially attributed to under-recording, which is commonly found with repeated 24-HDRs [36,37], or overestimation, which has been found with the FFQ. FFQs tend to overestimate energy and nutrient intakes compared with other dietary assessment methods [7,8]. This overestimation may be related to the fact that the FFQ asks participants to recall the frequency of intake of a much larger number of food items listed in an FFQ than an open-ended 24-HDR [31]. Furthermore, the high estimation of fiber intake in our study may be related to the over-reporting of fruit and vegetable consumption by subjects seeking social approval, which has been previously reported as a common bias [38]. Diabetes patients are often encouraged to consume more nutrient-dense foods such as whole grains, vegetables, and fruits. Hoping to show that they are trying to take care of themselves, patients with diabetes may over-report their consumption of fruit and vegetables to their nurses or nutritionists, which could partially explain the over-estimation of fiber intakes assessed by an FFQ.

It is rare that studies explain their reasons for using FFQs to assess intakes. In a review assessing 223 FFQs, it was estimated that 52% (115/223) of FFQs they reviewed were designed to assess intakes of specific foods or food groups, and 74% (166/223) were designed assess intakes of different nutrients [8]. Because our FFQ was intended for use in a busy outpatient medical setting where time is limited, we designed it to only ask 45 food intake questions. Thirty-three of the questions were about food groups rather than single food items. FFQs published from 1980 and 1999 ranged from 5 to 350 items (median 79 items) [8]. Four recent FFQs published in Taiwan contain 37–64 items [20,21,22,23]. Some recently developed FFQs have 164 [9], 146 [32], and 91 items [39] for Caucasian adults, and one FFQ has 98 items for adult diabetes patients in Brazil [11]. Furthermore, according to Willett in his book *Nutritional Epidemiology* [7], increasing the number of questions in an FFQ comes with only a marginal gain in useful information. Citing one study comparing a 44-item FFQ with a 273-item FFQ, Willet found that little was gained by increasing the item numbers. Therefore, it would be safe to assume that our 45-food FFQ can satisfactorily assess macronutrient intakes of our diabetes population.

Use of dietary biomarkers to further measure certain nutrients or food groups can be of value because no dietary assessment is free of error [7,8]. We measured three additional biomarkers (α-carotene, β-carotene, lutein) not evaluated by the three 24-HDRs to help us assess the performance of our FFQ. This may provide useful information because certain food groups can be affected by many biological factors including bioavailability, metabolism, and genetic factors other than intake itself [40]. Our previous investigations have shown that high fat, high meat, and high fish-vegetable dietary patterns analyzed by factor analysis of data obtained from a similar FFQ were significantly associated with blood ferritin [25] and with some n-3 polyunsaturates [41] in patients with type 2 diabetes. In univariate analysis, this study found a positive and significant correlation between increased intake of vegetables (including light and dark vegetables) and plasma α- and β-carotene concentrations, and negative associations between intake of fried food, egg products, and fermented products and plasma levels of both carotenes. These findings are fairly consistent with investigations showing that intakes of fruits and vegetables as measured by FFQ are good predictors of certain plasma carotenoids, including α-carotene, β-carotene, lycopene, and cryptoxanthin in diverse populations [42,43,44]). Furthermore, our finding that some unhealthy food groups (fried foods, fermented products) were negatively associated with α-carotene was similar to a recent study conducted in 361 Norwegian postmenopausal women [45]. The study reported that the healthy dietary pattern in their study (vegetarian pattern) positively correlated with six plasma carotenoids, whereas the unhealthy dietary patterns in their study (Western and continental patterns) were negatively related to some carotenoids [45]. Additionally, the absorption of carotenoids from a meal can be affected by various factors, including the food matrix [46]. Eggs are a highly bioavailable source of lutein and zeaxanthin because of their incorporation in the lipid matrix of the yolks [47]. Recent human intervention trials have reported that consumption of 1.3 eggs for 4.5 weeks in hypercholesterolemic men [48], three whole eggs for 12 weeks in adults with metabolic syndrome [49], and two eggs for four weeks in healthy adults [50] all resulted in elevation of plasma lutein and zeaxanthin. These human supplementation studies confirmed that eggs may represent an important food source for enhancing plasma carotenoids. However, in our study, egg consumption did not appear related to plasma lutein levels. This lack of correlation may be partially attributed to the fact that we only applied a FFQ administered to our subjects to recall status of egg consumption over the past year, but did not actually have our subjects supplement their diets with a fixed number of eggs.

Additionally, based on regression of backward elimination, we did not find that light- or dark-colored vegetables were separately correlated with lutein, only that total light- and dark-colored vegetables together were marginally correlated. Although increased tertile levels of light- or dark-colored vegetable consumption showed elevating trends in lutein, they were not significant. This lack of correlation with lutein can be partially attributed to the fact that our 45-item FFQ contained only four questions concerning the intake of vegetables and fruits as food groups without specifying individual items. For example, individually, green vegetables such as parsley, spinach, chicory, chard, broccoli, zucchini, and peas are reported to be rich in lutein [51], but we did not find intake of “dark- and light-vegetables” as a food group to be associated with lutein. Another reason may be that, unlike beta-carotene, lutein cannot be converted in the body into vitamin A (retinol). Yet another reason for the marginality of the correlation may be due to the small number of participants for whom we performed the carotenoid analysis.

This study has some other limitations. One limitation is that only a small number of subjects participated in this FFQ validation study and we covered only a limited number of food items in our FFQ. Another limitation is that we did not test reproducibility of our FFQ data. Long-term reproducibility of FFQ should be evaluated again within at least one year. Still another limitation is that we only assessed three carotenoids as biomarkers, this may limit to confirm intakes of fruit and vegetables. Future studies should be performed using other biomarkers to confirm more nutrients or specific food groups, such as urinary urea-N for protein intakes, blood fatty acids for foods containing different classes of fats, or biomarkers for micronutrients.

## 5. Conclusions

In conclusion, this short 45-item FFQ proved to be a valid assessment of energy, macronutrient, and fiber intake from diets usually consumed by patients with type 2 diabetes. Consumption of vegetables corresponds with plasma concentrations of α-carotene and β-carotene. These findings suggest that the FFQ could potentially be used in busy clinical settings treating patients with type 2 diabetes in Asia or diabetes patients consuming their usual diets.

## Figures and Tables

**Table 1:** Differences and correlation between intakes of nutrients obtained using the food frequency questionnaire (FFQ) and those obtained using three 24-h dietary recalls (24-HDRs) (*n* = 126).

Nutrient		FFQ ^1^	Three 24-HDRs ^1^	% Difference ^2^	*p*-Value ^3^	Pearson Correlation Coefficient(*r*) ^4^	
						Crude Data ^5^	Adjusted ^6^
Energy	kcal/day	1780.2 ± 654.0	1712.2 ± 466.1	6.2	0.593	0.501 **	-
Protein	g/day	61.3 ± 22.7	64.1 ± 23.6	−0.1	0.065	0.574 **	0.651 **
Fat	g/day	73.3 ± 32.5	67.3 ± 36.9	19.7	0.025	0.514 **	0.587 **
Carbohydrate	g/day	219.0 ± 102.4	226.3 ± 71.2	−0.7	0.020	0.438 **	0.639 **
Fiber	g/day	18.4 ± 10.4	16.2 ± 8.1	20.1	0.015	0.666 **	0.664 **

^1^ Nutrient intakes derived from the three 24-HDRs and the FFQ were expressed as mean ± SD. ^2^ %difference = (FFQ) − (Three 24-HDRs)/Three 24-HDRs × 100. ^3^ *p*-value: paired *t* test was used to test nutrient intake differences between the FFQ and the Three 24-HDR. ^4^ The energy and nutrient values were log-transformed to normalize the distribution, and the correlation coefficients were calculated. ^5^ Crude data were determined by using the Pearson correlation coefficient. ^6^ Model was adjusted for energy intake using the residual method, and partial correlation coefficient was determined. ** significance at *p* < 0.001.

**Table 2:** Cross-classification of daily energy-adjusted macronutrient intake based on the short food frequency questionnaire (FFQ) and the three 24-h dietary recalls (24-HDRs) (*n* = 126).

Nutrient	Cross-Classification (%)			
	Same Quartile	Adjacent Quartile	One Quartile Apart	Extreme Quartile
Protein (g/day)	39.7	33.3	22.2	4.8
Fat (g/day)	42.1	29.4	20.6	7.9
Carbohydrate (g/day)	40.5	34.1	16.7	8.7
Fiber (g/day)	40.5	40.5	17.5	1.6

Data is presented as % of participants categorized into the same, adjacent, one-quartile-apart and extreme quartiles. Nutrient intakes were log-transformed to fit the normality.

**Table 3:** Various plasma carotenoid concentrations analyzed by tertile intake levels of selected food categories used in the food frequency questionnaire (FFQ) (*n* = 71) ^1^.

Food Groups	α-Carotene (μg/dL)	β-Carotene (μg/dL)	Lutein (μg/mL)
Fried Food (T/W)			
Tertile 1	9.61 ± 8.09	42.14 ± 29.64	29.50 ± 17.28
Tertile 2	7.59 ± 5.31	37.48 ± 28.63	25.70 ± 11.29
Tertile 3	5.72 ± 3.74	28.78 ± 16.25	27.58 ± 14.19
*p* for trend ^2^	0.022	0.058	0.655
Eggs (P/W)			
Tertile 1	11.71 ± 9.57	47.37 ± 23.66	33.48 ± 16.27
Tertile 2	7.36 ± 4.76	35.07 ± 25.01	24.10 ± 10.12
Tertile 3	6.29 ± 4.86	32.23 ± 25.66	28.19 ± 15.60
*p* for trend ^2^	0.012	0.100	0.570
Marine fish (T/W)			
Tertile 1	5.28 ± 3.64	28.82 ± 14.46	24.95 ± 13.34
Tertile 2	7.04 ± 5.62	33.12 ± 26.03	28.73 ± 14.30
Tertile 3	9.97 ± 7.40	44.30 ± 28.72	28.67 ± 15.84
*p* for trend ^2^	0.010	0.041	0.428
Light-colored vegetables (P/W)			
Tertile 1	4.89 ± 2.55	26.54 ± 12.71	24.44 ± 10.59
Tertile 2	8.16 ± 5.85	37.89 ± 29.80	29.45 ± 15.94
Tertile 3	9.12 ± 7.30	41.09 ± 28.56	29.18 ± 16.10
*p* for trend ^2^	0.011	0.036	0.247
Dark-colored vegetables (P/W)			
Tertile 1	4.82 ± 2.59	25.69 ± 12.65	23.42 ± 10.63
Tertile 2	8.29 ± 5.69	41.46 ± 29.24	29.10 ± 15.94
Tertile 3	9.12 ± 7.32	40.20 ± 28.56	30.02 ± 15.83
*p* for trend ^2^	0.009	0.041	0.100
Fresh fruits (P/W)			
Tertile 1	5.60 ± 4.20	32.88 ± 20.41	22.16 ± 11.24
Tertile 2	7.96 ± 6.92	32.29 ± 21.10	28.97 ± 14.38
Tertile 3	7.89 ± 5.78	40.53 ± 30.68	28.44 ± 15.61
*p* for trend ^2^	0.397	0.262	0.342
Fermented products (T/W)			
Tertile 1	9.12 ± 7.04	39.87 ± 26.84	29.45 ± 15.07
Tertile 2	8.15 ± 4.99	31.63 ± 9.77	28.21 ± 23.39
Tertile 3	6.07 ± 5.10	32.95 ± 26.19	26.05 ± 11.85
*p* for trend ^2^	0.044	0.283	0.350
Fruit juice (T/W)			
Yes	7.69 ± 6.31	34.26 ± 23.06	27.99 ± 15.07
No	6.88 ± 4.89	43.84 ± 35.76	26.06 ± 11.17
*p* ^3^	0.690	0.253	0.687

Abbreviations: T/W: times per week; P/W: portions per week. ^1^ Data is expressed as mean ± SD and *p* < 0.05 was considered significantly different. ^2^ Simple linear regression was used to assess trends of carotenoids concentrations against tertile levels of FFQ food categories. ^3^ The *t*-test was used to determine the differences between the two groups.

**Table 4:** Association between tertile intakes of vegetables based on the food frequency questionnaire (FFQ) and various carotenoids using backward selection multiple liner regression (*n* = 71) ^1^.

Model	Food Group	α-Carotene (μg/dL)			β-Carotene (μg/dL)			Lutein (μg/mL)		
		*β*	SE	*p*	*β*	SE	*p*	*β*	SE	*p*
Model 1 ^2^	Light-colored vegetables (P/W)	1.96	0.78	0.014	6.71	3.13	0.035	-	-	-
Model 2 ^2^	Dark-colored vegetables (P/W)	2.06	0.77	0.009	6.82	3.11	0.032	-	-	-
Model 3 ^2^	Dark- and light-colored vegetables (P/W)	1.82	0.85	0.037	9.52	3.30	0.005	3.38	1.96	0.090

Abbreviations: P/W: portions per week. ^1^ Data are expressed as beta (SE) and *p* < 0.05 was considered significantly different. ^2^ A backwards elimination method adjusting for age, gender, FFQ-derived total energy intake, diabetes duration, education, hemoglobin A1C (HbA1c; <6.5%, 6.5–9% and >9%), and use of vitamin supplements (multivitamins, vitamin A or vitamin C) was used to examine independent associations between tertile levels of vegetables and three carotenoid concentrations.

## Supplementary Materials

The following are available online at http://www.mdpi.com/1660-4601/15/6/1142/s1. Figure S1: Study protocol for BP4DM study, Table S1: Demographics and clinical characteristics of participants with Type 2 diabetes (*n* = 126), Table S2: The BP4DM study-Food Frequency Questionnaire: Food items, frequency and serving(s) of consumption over the past year.
