# Supplementary material for: Validity of a Short Food Frequency Questionnaire Assessing Macronutrient and Fiber Intakes in Patients of Han Chinese Descent with Type 2 Diabetes
Source: Int J Environ Res Public Health. 2018 Jun 1;15(6):1142. doi: 10.3390/ijerph15061142 (PMC6025601; doi:10.3390/ijerph15061142)
Supplement: Supplementary file 1 [file ijerph-15-01142-s001.pdf]

Table S1. Demographics and clinical characteristics of participants with Type 2 diabetes (n = 126).

Characteristics / Demographics Characteristics	Overall
Age (years)	
<65	67 (53.2)
≥65	59 (46.8)
Diabetes duration at recruitment (years)	
<10	60 (47.6)
≥10	37 (29.4)
Missing data	29 (23.0)
Education years (years)	
< high school	30 (23.8)
≥ high school	66 (52.4)
Missing data	30 (23.8)
Smoking status	
Non-smoker	25 (19.8)
Ex-smoker	83 (65.9)
Current smoker	18 (14.3)
Eating out	
≤1–2 times/week	51 (40.5)
3–6 times/week	9 (7.1)
≥1 times/day	66 (52.4)
Anthropometric measure	
Body mass index (kg/m^2)	27.0 ± 4.1
Waist circumferences (cm)	92.3 ± 10.7
Clinical examination	
Systolic blood pressure (mmHg)	124.0 ± 76.1
Diastolic blood pressure (mmHg)	65.5 ± 71.8
Triglycerides (mg/dl)	164.8 ± 228.5
Total cholesterol (mg/dl)	167.9 ± 30.4
HDL-C (mg/dl)	46.2 ± 14.6
LDL-C (mg/dl)	92.0 ± 24.4
Fasting plasma glucose (mg/dl)	139.6 ± 45.4
HbA1c Glycated hemoglobin (%)	7.0 ± 1.0
<6.5	48 (38.4)
6.5–9	69 (55.2)
>9	8 (6.4)
Blood Urea Nitrogen (mg/dl)	15.7 ± 5.8
Serum creatinine (mg/dl)	0.9 ± 0.3
Albumin creatinine ratio (ACR, mg/gm)	34.4 ± 54.4

Abbreviations: HDL: High-density lipoprotein; LDL: Low-density lipoprotein; HbA1c: Glycated hemoglobin. Data are expressed as n (%) or mean ± SD.

Table S2. The BP4DM study-Food Frequency Questionnaire: Food items, frequency and serving(s) of consumption over the past year.

**Food Items**	**Frequency of Food Intake**									**Amount**
	**Times/Month**			**Times/Week**			**Times/Day**			**Servings per Time**
	**never**	**≤1**	**2–3**	**1–2**	**3–4**	**5–6**	**1**	**2–3**	**4–6**	**0~7 ***
**1.Fatty meats and skins** (e.g., pork belly, chicken skin, fish skin, duck skin)	☐	☐	☐	☐	☐	☐	☐	☐	☐	

Food Items	Frequency of Food Intake									Amount
	Times/Month			Times/Week			Times/Day			Servings per Time
	never	≤1	2–3	1–2	3–4	5–6	1	2–3	4–6	0~7 *
**2.Deep fried foods** (e.g., fried pork or chicken cutlets, fried chicken pieces, fried fish and tofu, donuts)	☐	☐	☐	☐	☐	☐	☐	☐	☐	
**3.Eggs** (chicken, duck, quail, etc.)<br>1 serving = one chicken or duck egg, five quail eggs or two chicken egg whites	☐	☐	☐	☐	☐	☐	☐	☐	☐	☐
**4.Seafood** (e.g., prawns, clams, squid, crabs, oysters, shrimp etc.)	☐	☐	☐	☐	☐	☐	☐	☐	☐	
**5.Marine fish** (e.g., Spanish mackerel, mackerel pike, salmon, ribbon fish, tuna, snapper, etc.)<br>1 serving = 35 grams of fish meat, about 3 female fingers wide and 1 finger thick	☐	☐	☐	☐	☐	☐	☐	☐	☐	☐
**6.Freshwater fish** (e.g., milk fish or white mullet, tilapia, eel, etc.)<br>1 serving = 35 grams of fish meat, about 3 female fingers wide and 1 finger thick	☐	☐	☐	☐	☐	☐	☐	☐	☐	
**7.Percentage of fish deep fried**	**Approximately __________% fried**<br>**(0)0 (1)1~20 (2)21~40 (3)41~61 (4)61~80 (5)81~100**									
**8.Red meats** (e.g., pork, beef, lamb)<br>1 serving = 35 grams of fish meat, about 3 female fingers wide and 1 finger thick	☐	☐	☐	☐	☐	☐	☐	☐	☐	☐
**9.White meats** (e.g., chicken, duck, goose, poultry)<br>1 serving = 35 grams of meat, about three female fingers wide and 1 finger thick, or two wings, one thigh or one drumstick	☐	☐	☐	☐	☐	☐	☐	☐	☐	
**10.Percentage of meat deep fried.**	Approximately __________% fried<br>(0)0 (1)1~20 (2)21~40 (3)41~61 (4)61~80 (5)81~100									
**11.Canned meats** (e.g., tuna, eel, crab, ground meat, dried shredded meats)	☐	☐	☐	☐	☐	☐	☐	☐	☐	
**12.Processed meats** (e.g., ham, sausage, preserved meat, bacon, hot dog, salty pork, smoked meats, etc.)	☐	☐	☐	☐	☐	☐	☐	☐	☐	
**13.Milk and yogurt** (e.g., cow milk, goat milk, yogurt)<br>1 serving = 240c.c., 3-4 tablespoons milk powder, 200c.c. liquid yogurt, 100 c.c thick regular yoghurt	☐	☐	☐	☐	☐	☐	☐	☐	☐	☐
**14.Processed dairy products** (e.g., block cheeses, creamed cheeses, ice cream, etc.)	☐	☐	☐	☐	☐	☐	☐	☐	☐	☐

Food Items	Frequency of Food Intake									Amount
	Times/Month			Times/Week			Times/Day			Servings per Time
	never	≤1	2–3	1–2	3–4	5–6	1	2–3	4–6	0~7 *
1 serving = 2 cheese slices, 1 medium scoop ice cream, 240 c.c. flavored milk, etc.										
**15.Soy products** (e.g., dried tofu, fresh tofu, soy pudding, soy milk, bean curd, etc.)<br>1 serving = 35 grams cm. x cm dried tofu, 80 – 100 grams fresh tofu or bean curd, or a half container, 240c.c. unsweetened soy milk	☐	☐	☐	☐	☐	☐	☐	☐	☐	☐
**16.Processed wheat or gluten products** (e.g., fried or roasted gluten, gluten flour, vegetable protein or products)	☐	☐	☐	☐	☐	☐	☐	☐	☐	
**17.Light-coloured vegetables** (e.g., cabbage, Chinese cabbage, Chinese radish, winter melon, loofah, etc.)<br>1 serving = about half rice bowl of cooked vegetables	☐	☐	☐	☐	☐	☐	☐	☐	☐	☐
**18.Dark-coloured vegetable**s (e.g., broccoli, spinach, mushroom, Chinese green vegetables, carrot, etc.)<br>1 serving = half bowl of vegetables cooked	☐	☐	☐	☐	☐	☐	☐	☐	☐	
**19.Percentage of vegetables cooked in oil** (usually sautéed or stir-fried)	**Approximately __________% sautéed or stir-fried**<br>**(0)0 (1)1~20 (2)21~40 (3)41~61 (4)61~80 (5)81~100**									
**20.Pickled vegetables** (e.g., pickled cucumbers, cabbage, dried bamboo shoots, radishes, kimchi, etc.)	☐	☐	☐	☐	☐	☐	☐	☐	☐	
**21.Fresh fruit**<br>1 serving = a portion fruit about the size of a female fist or 3/4 a rice bowl (one cup)	☐	☐	☐	☐	☐	☐	☐	☐	☐	☐
**22.Sweetened fruit juice** (e.g., bottled juice or fresh juice with sugar, condensed juice, coconut water) 1 serving = 120-150c.c.	☐	☐	☐	☐	☐	☐	☐	☐	☐	
**23.Sugar-containing packaged beverages** (e.g., coffee and tea with sugar, milk tea, soy milk with sugar, sports drinks, energy drinks, soft drinks, yakult)	☐	☐	☐	☐	☐	☐	☐	☐	☐	
**24.Sugarless Tea** (e.g., oolong, black (red), pu-er, green)	☐	☐	☐	☐	☐	☐	☐	☐	☐	
**25.Chinese staples** (e.g., rice, porridge, noodles, rice noodles, steamed buns)<br>1 serving = one Chinese bowl of rice, two Chinese bowls porridge or rice noodles, one steamed bun	☐	☐	☐	☐	☐	☐	☐	☐	☐	☐

Food Items	Frequency of Food Intake									Amount
	Times/Month			Times/Week			Times/Day			Servings per Time
	never	≤1	2–3	1–2	3–4	5–6	1	2–3	4–6	0~7 *
**26.Percentage of staples cooked in oil** (sautéed or stir fried)	**Approximately __________% sautéed or stir fried**<br>**(0)0 (1)1~20 (2)21~40 (3)41~61 (4)61~80 (5)81~100**									
**27.Bread and bun** (e.g., white bread slice, soft roll, croissant, sweet bun, red bean bun)<br>1 serving = 2 slices of white bread, one soft roll, two croissants, one sweet bun, one red bean bun	☐	☐	☐	☐	☐	☐	☐	☐	☐	☐
**28.Root vegetables** (e.g., sweet potatoes, potatoes, taro, water caltrop, lotus root, Chinese yam)<br>1 serving = about 4 wedges or a half Chinese rice bowl	☐	☐	☐	☐	☐	☐	☐	☐	☐	☐
**29.Low-nitrogen staple foods** (e.g., bean noodles, pearl sago/tapioca, tapioca starch products)	☐	☐	☐	☐	☐	☐	☐	☐	☐	
**30.Starchy/thickened soup and foods using a starch thickeners** (e.g., porridge, soup thickened with corn starch or tapioca flour)	☐	☐	☐	☐	☐	☐	☐	☐	☐	
**31.Cakes and cookies** (e.g., sponge cakes, pineapple jam cookies, walnut cookie, other cookies cookies)	☐	☐	☐	☐	☐	☐	☐	☐	☐	
**32.Seeds and nuts** (e.g., peanuts, pumpkin seeds, pistachios, almonds, walnuts, cashews, pine nuts, sesame seeds)	☐	☐	☐	☐	☐	☐	☐	☐	☐	
**33.Eating outside** (a restaurant, takeaway)	☐	☐	☐	☐	☐	☐	☐	☐	☐	
**34. Use of sugar substitute** (e.g., Zero Coke and other 0 calories soft drinks, sugar-free candy or chewing gum, sorbitol, oligo-fructose)	☐	☐	☐	☐	☐	☐	☐	☐	☐	
**35.Low calorie desserts and snacks** (e.g., konjac, gelatin, white fungus, agar dessert, seaweed nori, etc.)	☐	☐	☐	☐	☐	☐	☐	☐	☐	
**36.Hot foods and liquids served using styrofoam or plastic cups**	☐	☐	☐	☐	☐	☐	☐	☐	☐	
**37.Hot foods packaged using plastic bags**	☐	☐	☐	☐	☐	☐	☐	☐	☐	
**38.Hot food served on disposable dishes**	☐	☐	☐	☐	☐	☐	☐	☐	☐	
**39.Handmade drinks** (e.g., fruit juices, teas, sometime with gelatinous candies added)	☐	☐	☐	☐	☐	☐	☐	☐	☐	
**40.Cup size of handmade drinks**<br>1 serving = one cup	**☐ Do not eat ☐ 360 c.c (small) ☐ 500 c.c (medium)**<br>**☐ 750 c.c (large) ☐ 1000 c.c (extra large)**									
**41.Sweetness level of handmade drinks**	**☐ Sugar-free ☐ Quarter sugar ☐ Half sugar**<br>**☐ Less sugar ☐ Regular sugar**									

Food Items	Frequency of Food Intake									Amount
	Times/Month			Times/Week			Times/Day			Servings per Time
	never	≤1	2–3	1–2	3–4	5–6	1	2–3	4–6	0~7 *
**42.Sauces use** (e.g., sweet-chili pepper sauce, soy sauce, chili pepper sauce, thick broad-bean sauce, BB sauce)	☐	☐	☐	☐	☐	☐	☐	☐	☐	
**43.Fermented soy processed products** (e.g., vegetarian ham, stinky tofu, jujube, and other vegetarian meats)	☐	☐	☐	☐	☐	☐	☐	☐	☐	
**44.Fermented foods** (e.g., fermented bean curd, fermented soya beans, miso, natrium bean)	☐	☐	☐	☐	☐	☐	☐	☐	☐	
***Answer how many servings (0, 1, 2, 3, 4, 5, 6, 7) consumed per time.**										

45. Dietary Supplement Use: Over the past six months, have you used the following dietary supplements?

Dietary Supplement Products	Usage Frequency			Usage# time(s)	Usage+ Amount	Usage Unit			
	Month	Week	Day			Pill	Bottle	Package	Other
**Multivitamin**									
**Vitamin A**									
**Vitamin B complex**									
**Vitamin C**									
**Calcium**									
**Fish oil**									
**Glucosamine**									
**Energy Drink**									
**High-protein powder**									
**Low protein powder**									
**Nutrition formula for diabetes**									
**Probiotics**									
**Other**									
# Answer number of times used at particular frequency (month, week or day)									
+ Answer amount used each time									

[1] This food frequency questionnaire was developed for type 2 diabetes patients enrolled in an ongoing intervention study investigating the effect of tight blood pressure control on reduction of renal risk for type 2 diabetes mellitus (the BP4DM study Clinicaltrials.gov NCT03477786).

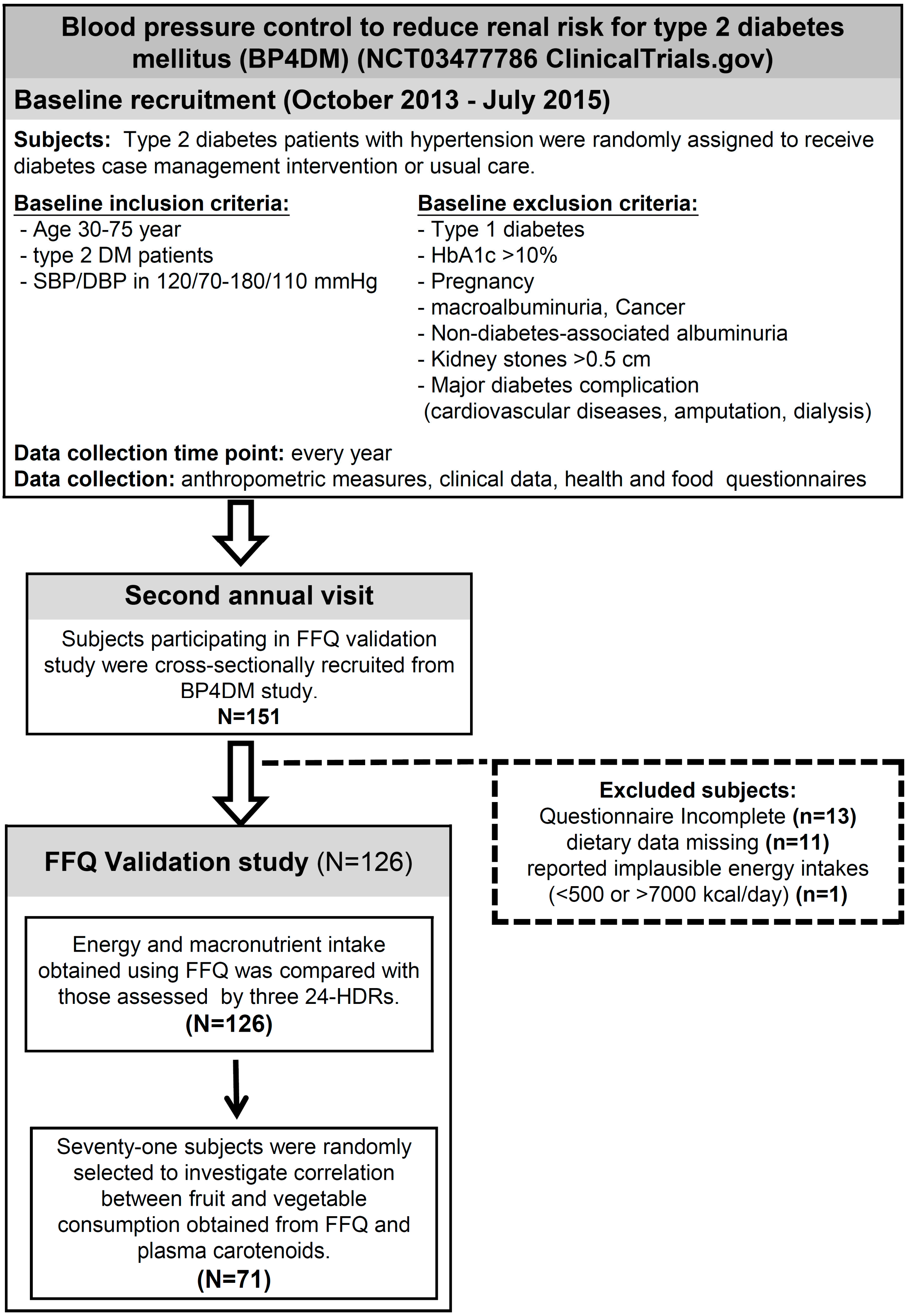

Figure S1. Study protocol for BP4DM study.

The study subjects were chosen from a cohort of type 2 diabetes patients enrolled in a clinical trial to examine the effect of tight blood pressure control on reduction of renal risk for type 2 diabetes mellitus (the BP4DM study Clinicaltrials.gov NCT03477786). The original study recruited 30- to 75-year-old type 2 diabetes patients with hypertension (systolic or diastolic blood pressure > 140/90 mmHg) registered to participate in a multidisciplinary diabetes shared-care program. Subjects not eligible at baseline recruitment included those with type 1 diabetes, gestational diabetes, pregnant women, hemoglobin A1c (HbA1c) >10%, eGFR <30 ml/min/1.73 m2, albuminuria >

300 mg/g, presence of non-diabetes-associated albuminuria, kidney stones >0.5 cm, or a history of myocardial infarction as well as those with a history of cerebrovascular events, foot amputation, dialysis, cirrhosis or cancer under active treatment within 3 years. We cross-sectionally recruited 150 of the subjects participating for the second annual visit and 25 subjects were excluded due to missing or incomplete dietary data and implausible energy intakes.
